# Coronary microvascular dysfunction in childhood: An emerging pathological entity and its clinical implications

**DOI:** 10.1016/j.ahjo.2024.100392

**Published:** 2024-04-12

**Authors:** Takeshi Tsuda, Gina Patel

**Affiliations:** a Nemours Cardiac Center, Nemours Children's Health, Wilmington, DE 19803, USA; b Department of Pediatrics, Sidney Kimmel Medical College of Thomas Jefferson University, Philadelphia, PA 19107, USA

**Keywords:** Coronary vasculature, Myocardial ischemia, Inflammation, Endothelial dysfunction, Coronary flow reserve, Cardiomyopathy

## Abstract

Coronary microvascular dysfunction (CMD) encompasses a spectrum of structural and functional alterations in coronary microvasculature resulting in impaired coronary blood flow and consequent myocardial ischemia without obstruction in epicardial coronary artery. The pathogenesis of CMD is complex involving both functional and structural alteration in the coronary microcirculation. In adults, CMD is predominantly discussed in context with anginal chest pain or existing ischemic heart disease and its risk factors. The presence of CMD suggests increased risk of adverse cardiovascular events independent of coronary atherosclerosis. Coronary microvascular dysfunction is also known in children but is rarely recognized due to paucity of concommitent coronary artery disease. Thus, its clinical presentation, underlying mechanism of impaired microcirculation, and prognostic significance are poorly understood. In this review article, we will overview variable CMD reported in children and delineate its emerging clinical significance.

## 1 Introduction

Abnormal coronary microcirculation has emerged as an important clinical entity in adults with anginal chest pain and myocardial ischemia with no identifiable epicardial coronary artery obstruction [1,2]. In adults, coronary microvascular dysfunction (CMD) predominantly occurs in context with coronary syndrome (ACS) or symptomatic angina and is associated with increased mortality and incidence of major adverse cardiac events [3]. The underlying pathophysiology of CMD is multifactorial, encompassing impaired vasodilatation, increased vasoconstriction, and inward remodeling in coronary microvasculature [4]. Although the presence of CMD likely indicates detrimental prognosis, there has been no known effective treatment to reverse or attenuate this pathological process.

Although rare, CMD can occur in children mostly due to a primary coronary vascular endothelial impairment rather than in relation to existing coronary atherosclerosis; adult-type coronary artery disease (CAD) is rare in children. In children, acute myocardial ischemia or infarction can occur in congenital or acquired coronary artery diseases that differ from adult coronary artery disease [5]. In fact, CMD has been most intensively studied in Kawasaki disease (KD) [[6], [7], [8], [9], [10]], one of a few diseases that can cause myocardial infarction (MI) in children. Because of the distinct difference between adults and children regarding clinical background responsible for myocardial ischemia, clinical significance and prognostic implication of CMD may be different in children when compared with adults.

In this article, we will review underlying pathological mechanisms of CMD in children, propose a unique classification of CMD based upon the pathophysiology, and discuss its clinical implications.

## 2 Clinical significance of coronary microvasculature circulation in relation to cardiovascular diseases

The coronary microvascular network plays a pivotal role in regulating blood flow distribution within working myocardium. The coronary vascular system consists of three components with different functions; large epicardial arteries (approximately 500 μm to 2 to 5 mm), periarterioles (approximately 100 to 500 μm), and intramural arterioles (<100 μm), each with different physiological roles [11]. Large epicardial coronary arteries assume a capacitance function with little resistance to coronary blood flow. Periarterioles generate measurable pressure difference along their length but are not under direct vasomotor control by diffusible myocardial metabolites because of their extramyocardial position wall thickness. The intramural arterioles are characterized by their considerable drop in pressure along their path; their function is to match the myocardial blood supply and oxygen consumption [11].

Coronary microvascular control is orchestrated by multiple factors: 1) physical forces (perpendicular stress caused by blood pressure and shear stress in the longitudinal direction, 2) metabolic factors (myocardial oxygen consumption, autoregulation, reactive hyperemia caused by focal ischemia), and 3) neurohormonal factors (neurotransmitters, circulating vasoactive hormones) [12]. Importantly, coronary microcirculation represents >90 % of the whole coronary circulation and is the primary gatekeeper for myocardial blood flow beyond the epicardial coronary arteries [13]. Following immediate changes in vascular tone (seconds to minutes) in response to the above factors, more chronic structural vascular adaptations occur (days-weeks-months), resulting in enlargement of caliper of vessels (vascular remodeling), collateralization (arteriogenesis), and angiogenesis as dynamic vascular adaptation [14,15].

Coronary microvascular dysfunction can be defined as a suboptimal coronary vasodilator response to exercise or pharmacological stress. In clinical practice, coronary flow reserve (CFR) is regarded as a marker to assess microvascular function [16]. For diagnosis of CMD, demonstration of abnormal coronary vascular responses to exercise or pharmacological stress and the reproduction of symptoms are considered essential. Mechanisms of alteration in coronary blood flow are classified into three categories, including extravascular factors (increased myocardial metabolic demand, external compression, and decreased diastolic perfusion time), vascular dysfunction (endothelial dysfunction, vascular smooth muscle cell dysfunction, and altered autonomic nervous system), and vaso-structural changes (obstruction, infiltration, remodeling, rarefaction, and perivascular fibrosis) [17].

Crea and his colleagues proposed a classification of a spectrum of CMD into four groups according to different clinical scenarios: 1) CMD in patients without obstructive CAD, myocardial disease and valvular heart disease, 2) CMD in patients with obstructive CAD, 3) CMD in patients with myocardial disease and valvular heart disease, and 4) iatrogenic CMD [4,11,18]. These patients tend to present with symptoms of ischemic heart disease (IHD) and/or its risk factors, including diabetes mellitus (DM), hypertension, smoking, dyslipidemia, or obesity. As acute coronary syndrome is quite uncommon in children, this classification may not appropriately represent CMD in the pediatric population. In addition to the above four categories, Herrmann et al. proposed an additional subtype (“after cardiac transplant”), in which CMD is mediated by alteration in autonomic tone, inflammation and immune mechanisms, and possibly defective endothelial progenitor cell recruitment [17]. Involvement of CMD has been reported in some types of congenital heart disease (CHD), mostly without clinical signs of ACS, which is seldom discussed in adults and is under-recognized [19]. This heterogeneity of CMD needs to be recognized when discussing clinical significance of CMD, especially in children.

## 3 Coronary microvascular dysfunction in pediatric population

Based upon the reported incidence and the underlying pathophysiology of CMD in children, we have classified CMD into the following four categories: I. Primary coronary vascular impairment/vascular inflammation, II. Structural heart disease and structurally abnormal coronary microvasculature, III. Hemodynamically induced myocardial supply and demand mismatch, and IV. Miscellaneous (Table 1).

**Table 1:** Classification of CMD in children.

I. Primary coronary vascular impairment/vasculitis/inflammation a. Kawasaki disease (KD) b. Infection or post-infectious (COVID 19) c. Autoimmune/rhematic disorders d. Post-heart transplant (cardiac allograft vasculopathy) e. Drug-induced f. Risk factors of ischemic heart disease (diabetes mellitus, hypertension, dyslipidemia, obesity, and smoking) II. Structural heart disuse a. Cardiomyopathies 1) Hypertrophic cardiomyopathy (HCM) 2) Dilated cardiomyopathy (DCM) b. Congenital heart disease with congenital coronary anomalies 1) Transposition of the great arteries (TGA) 2) Single ventricle 3) Anomalous left coronary artery from pulmonary artery (ALCAPA) 4) Pulmonary atresia with intact ventricular septum (PA/IVS) III. Hemodynamically induced myocardial oxygen supply and demand mismatch a. Severe aortic valve stenosis b. Hypertensive cardiomyopathy IV. Miscellaneous

### 3.1 Primary coronary vascular impairment

#### 3.1.1 Kawasaki disease

Kawasaki disease (KD) is a multisystemic vasculitis frequently encountered in infants and children. The etiology of KD is poorly understood, but it is known to cause coronary artery aneurysms in 15 % to 25 % of untreated children making it the most common acquired heart disease in children. Acute vasculitis associated with KD may be responsible for the development of a complex set of coronary artery abnormalities, from epicardial coronary arteries to microvasculature, with vascular endothelial damages and dysfunction [10]. Coronary artery aneurysms increase the mortality and morbidity by increasing the risk of MI, ischemic heart disease, and sudden cardiac death [20].

In KD, coronary arteritis begins at 6 to 8 days after the onset; the inflammation rapidly involves all layers of the artery, characterized by infiltration of monocytes and macrophages [21]. One of the earliest electron microscopic evaluations of endomyocardial biopsies of the right ventricle in 10 patients with KD demonstrated regional intracellular edema with increased glycogen granule deposition in the sarcoplasm. Further, myocardial cells were occasionally noted to have loss of myofibrils and loss of sarcomere structures. The biopsies during the active phase of the disease demonstrated more prominent abnormalities, which suggested cardiac involvement could be subdivided into myocardial ischemia due to coronary arteritis and myocarditis/pericarditis. The regional distribution of the histopathologic changes in the myocardium were attributed to ischemic changes from vasculopathy of the arterioles and small arteries [22].

Coronary artery lesions have mostly been studied on autopsy specimens. Liu et al. studied the right ventricular endomyocardial biopsy specimens in the follow up of 54 KD patients with and without coronary artery lesions [8]. They identified histopathologic changes consistent with myocardial hypertrophy, myofibril degeneration and disarray, inflammation, fibrosis and microvascular lesions. The study confirmed prior findings of post-myocarditis changes, cardiomyopathy like changes and ischemic changes. Importantly, they found microvascular abnormalities in 72 % of the cohort, including microvascular dilation, thrombus formation, narrow or occlusive lumens, and thickened arteriolar walls. The incidence of microvascular lesions was more prevalent in KD patients with known coronary artery lesions than in those without [8]. Involvement of cardiomyocytes and microvasculature in KD was also reported in previous studies [6,7]. It is likely that myocardial ischemic events in KD are not solely due to abnormalities of large coronary arteries but also due to microvascular dysfunction.

Risk stratification is often difficult in KD patients especially when utilizing standard measurements with non-invasive imaging methods. Despite microscopic evidence of myocardial inflammation, standard echocardiographic measurements of left ventricular function are often normal. In addition, routine histopathologic evaluation in KD follow up is often not feasible. Recent studies have reported other non-invasive imaging methods to better quantify myocardial work in KD patients. Sabatino et al. reported that other markers of myocardial work, myocardial work index, myocardial constrictive work, and myocardial work efficiency, were significantly reduced in KD with coronary artery dilation despite normal global longitudinal strain and function [23].

#### 3.1.2 Infections and post-infectious process

Severe cardiac complications were frequently observed in elderly COVID-19 patients and in those with chronic cardiovascular comorbidities including hypertension, DM, obesity, and existing heart disease. Pathogenesis of CMD in COVID-19 infection is complex, but characterized mainly by dysregulated immune system, overwhelming systemic inflammation, endothelial dysfunction, and coagulation abnormality resulting in microvascular embolism causing coronary microvascular obstruction [24], which is reportedly rare in children.

As a post-infectious process, multisystemic inflammatory syndrome in children (MIS-C) became a novel entity during the COVID-19 pandemic in 2020, consisting of post-viral myocarditis and inflammatory vasculopathy [25]. The syndrome shared many clinical characteristics with KD, including coronary artery aneurysm. In contrast with KD where vasculitis of large epicardial artery is a main feature of cardiac involvement, MIS-C has less coronary artery involvement and instead more global dysfunction, which is indicative of myocardial injury secondary to post-viral myocarditis [25,26].

Viral infections can have both direct and indirect effects on the endothelium. Despite COVID-19 geing predominantly a respiratory viral infection, there is a significant association with venous thromboembolisms. An autopsy study done in adult patients with COVID-19 to assess the risk of venous thromboembolism also demonstrated the presence of viral RNA at high concentrations in the heart and other organs in some of the patients indicating that the SARS-CoV-2 virus can spread in the blood stream [27]. It is still uncertain whether the endothelial dysfunction is due to direct effects of the SARS-CoV-2 virus or secondary to pro-inflammatory cytokines that are elevated in COVID-19 positive patients [25,28].

#### 3.1.3 Autoimmune disorders/rheumatic disorders

Systemic autoimmune diseases are disorders characterized by humoral and cell-mediated immune responses against various self-antigens. Inflammation-related CMD is a known entity that has been described in patients with rheumatologic diseases expressed either as an inability of microcirculation to dilate appropriately to meet myocardial oxygen demand or as coronary microvascular spasm. This type of CMD has been associated with increased incidence of ischemic heart disease, which contributes to high morbidity and mortality even in the absence of obstructive epicardial coronary artery disease [29,30]. Occult myocardial edema, myocarditis, diffuse subendocardial fibrosis, and MI are not unusual at the diagnosis of treatment-naïve rheumatic disorders, which are commonly unidentified by echocardiogram but detected by cardiac magnetic resonance imaging (MRI) [31].

Women diagnosed with rheumatoid arthritis (RA) have more than two-fold higher risk of MI, indicating that RA should be recognized as a maker for increased risk for MI [32]. Better control of RA activity is associated with reduction of cardiovascular disease, whereas the failure to achieve disease control aggravates the risk for subclinical atherosclerosis and cardiovascular disease (CVD) within one-year follow up, suggesting that baseline vascular inflammation is responsible for progression of both rheumatoid disorder and CVD [33].

Systemic lupus erythematosus (SLE) is also associated with premature atherosclerosis and increased cardiovascular risk; systemic inflammation that provokes CMD is demonstrated by high prevalence of coronary vasomotor abnormalities [34]. Multiple mechanisms secondary to immune dysregulation have been proposed to explain CMD and atherosclerosis in SLE, including oxidative stress, dysregulated cytokine cascade, neutrophil extracellular traps, autoantibodies, proinflammatory T cells, and compromised endothelial progenitor cells [35]. Just as in RA, SLE patients have at least two-fold increased incidence of cardiovascular complications with an estimated 10-year mortality rate of 26 % compared with that of 19 % for control subjects [36].

#### 3.1.4 Post-heart transplant (cardiac allograft microangiopathy)

Accelerated coronary arteriosclerosis is a complication of rejection reported in children and adults with heart transplants, called cardiac allograft vasculopathy (CAV), a leading cause of late mortality and allograft failure [37,38]. Accelerated coronary arteriosclerosis is characterized as a diffuse concentric process that affects both large and small coronary vessels. The underlying pathophysiology involves endothelial dysfunction secondary to non-immune or immune processes. Specifically, the presence of fibrointimal thickening of the arteries leads to progressive vascular obliteration, occlusive vascular changes, and subsequent organ ischemia [39].

The endothelium is a key regulator for vasomotor tone in large and small coronary vessels and the first area of exposure to immune mediated cells; it is, therefore, at risk for early immune injury. A prospective study looked at allograft biopsies in adult kidney transplant patients found that higher levels of circulating — donor-specific anti-HLA antibodies were correlated with major cardiovascular events by the triggering of cytokines and inflammatory mediators, which ultimately leads to generalized vascular injury [40]. The correlation of donor specific antibodies and cardiac vasculopathy has also been found in adults with heart transplants [41].

Furthermore, damage to the endothelium has been shown to affect its response to native vasodilators. Treasure et al. demonstrated that with time, the endothelium's ability to dilate deteriorates in adults after heart transplant increasing the risk of myocardial ischemia. It has been demonstrated that the microvasculature response to acetylcholine can gradually decrease during the initials years after heart transplants in adults [42]. Gagliardi et al. were among the first to study coronary microcirculation and its function after pediatric heart transplants and found that coronary blood flow did not increase with acetylcholine, an endothelium dependent vasodilator, indicating that there is endothelial damage of the coronary microcirculation [43]. Abnormal responses by graft coronary arteries to intensive immunosuppressive therapies may generate diffuse vasculopathy by dysregulating the intrinsic immune system.

#### 3.1.5 Drug-induced CMD

Certain drugs are known to adversely affect coronary microcirculation and compromise acute or long-term cardiovascular health.

Athletes with chronic abuse of anabolic steroids are known to develop adverse cardiovascular morbidity and all causes of mortality [44]. In 34 deaths of male uses of anabolic androgenic steroids in Sweden, chronic cardiac changes, myocardial hypertrophy and/or patchy fibrosis, were observed in 12 cases including two accidental acute poisonous cardiovascular deaths due to acute myocardial ischemia [45]. In the mouse model, Tagarakis et al. demonstrated that anabolic steroids impaired coronary microvascular adaptation to physical conditioning and was responsible for increased vulnerability to myocardial ischemia [46]. Other common misused drugs, including cannabis and cocaine, are also known to cause acute and chronic adverse cardiovascular effects, in part, by direct endothelial dysfunction causing MI via vasospasm of coronary microvasculature [47].

Anticancer drugs, especially anthracyclines, are known to cause acute and chronic cardiotoxicity [48,49]. The underlying mechanisms are multifold, but the main mechanism is thought to be inhibition of topoisomerase 2β resulting in activation of cell death pathways and inhibition of mitochondrial biogenesis, enhanced by cumulative anthracycline dosage, age, and preexisting cardiac disease [50]. Many forms of anti-cancer therapy induce endothelial damage including coronary microvasculature directory through primary effects on endothelial cells and indirectly through systemic effects such as innate immune system activation [51]. Using large-white male pigs, Galan-Arriola et al. demonstrated that intracoronary injection of anthracycline induced progressive and irreversible structural damages of coronary microcirculation independent of cardiac contractile deficits, suggesting an early occurrence of microvascular impairment even during subclinical stages of anthracycline-induced cardiotoxicity [52]. Vascular toxicity has emerged as an important consequence of cancer treatment that precedes development of cardiotoxicity and may contribute to the pathogenesis of cardiovascular complications in cancer survivors. Protecting the subclinical coronary microcirculation may become a new therapeutic target for preventing subsequent adverse cardiovascular events in cancer survivors with variable risk factors [51].

#### 3.1.6 Diabetes mellitus

Pathogenesis of diabetic cardiomyopathy is multifold, including cardiac insulin resistance, glucotoxicity, mitochondrial dysfunction, oxidative stress, endoplasmic reticulum stress, impaired calcium handling, and activation of systemic and local renin-angiotensin-aldosterone system [53]. Both micro- and macrovascular complications of long-standing Type 1 and 2 DM due to dysregulation of coronary endothelial cells and exosomes have recently emerged as important contributing factors of diabetic cardiomyopathy responsible for substantial mortality and morbidity [53]. It has been well studied that the underlying causes of coronary microangiopathy in DM are hyperglycemia and insulin resistance, which cause an imbalance between endogenous reactive oxygen species and nitric oxide. Furthermore, adult patients with Type 2 DM are at increased risk for having impaired microvascular dilation and thereby having reduced coronary flow reserve [29].

### 3.2 Structural heart disease

#### 3.2.1 Cardiomyopathies

##### 3.2.1.1 Hypertrophic cardiomyopathy

Hypertrophic cardiomyopathy (HCM) is the most common genetic myocardial disease with extreme heterogeneity and is the most common cause of sudden cardiac death (SCD) in the young, and it is a major cause of heart failure and disability at any age in association with myocardial ischemia and fibrosis [54]. Coronary angiopathy in patients with hypertrophic cardiomyopathy is well known; its pathogenesis has been linked to prominent structural abnormalities of small coronary arteries, such as intimal hyperplasia, medial hypertrophy and decreased luminal size in small intramural coronary arteries. In addition, physiological effects of extravascular compression due to myocardial hypertrophy, diastolic dysfunction, and LV outflow tract obstruction also occur [55,56]. The presence of coronary angiopathy in these patients increases the risk of myocardial ischemia. Furthermore, patients with genotype positive sarcomere myofilament mutations tend to have more severe coronary microangiopathy postulating that the sarcomere gene mutation causes adverse structural abnormalities in coronary microcirculation in these patients [54]. Cecchi et al. prospectively studied 51 patients with HCM over 8.7 ± 2.1 years (age 44 ± 13 years) and demonstrated that the response of myocardial blood flow to dipyridamole was severely blunted in HCM and also that the degree of CMD was a strong, independent predictor of clinical deterioration and death [57], underscoring the importance of recognition of subclinical CMD in HCM to predict prognosis and potentially to introduce early treatment. However, effective pharmacologic treatment of CMD has yet to be determined. Petersen et al. demonstrated that myocardial perfusion reserve was significantly reduced in HCM, particularly in the endocardium, in proportion to the magnitude of hypertrophy, and that ischemia was more prevalent and more severe in hypertrophied segments [58], supporting the previous observation that magnitude of hypertrophy is directly related to the risk of sudden death [59]. These studies suggest CMD as an important prognostic implication of life threatening adverse cardiovascular events in HCM.

##### 3.2.1.2 Dilated cardiomyopathy

Dilated cardiomyopathy (DCM) is characterized by reduced ventricular systolic function and dilatation of LV or LV and RV, resulting in symptomatic heart failure, functional disability, ventricular arrhythmias, and/or sudden cardiac death. In DCM patients without overt heart failure (NYHA class I or II), Neglia et al. showed that myocardial perfusion reserve was significantly impaired both at rest and in response to vasodilating stimuli when compared with the control patients and that the reduced perfusion was noted despite normal hemodynamics, suggesting intrinsic abnormality in coronary microvascular circulation in DCM [60]. Coronary microvascular dysfunction can be considered one of the pathogenic mechanisms involved in the evolution ventricular dysfunction towards heart failure possibly through progressive myocardial ischemic damage and may have an independent and relevant progressive value in the absence of epicardial coronary artery disease [61]. In addition, DCM increases myocardial oxygen demand due to increased wall stress, which consequently reduces the coronary flow reserve even further leading to worsening LV dysfunction [54].

#### 3.2.2 Congenital heart disease

The exact etiology, pathophysiology, and prognostic implications of CMD in individuals with congenital heart disease (CHD) are poorly understood. In CHD, CMD may be induced by either structural abnormalities (congenital structural abnormalities of the blood vessels, luminal obstruction, secondary vascular changes, or vascular rarefaction), functional abnormalities (endothelial dysfunction, dysfunctional vascular smooth muscle cells, or autonomic dysfunction) or extravascular mechanisms (extramural compression from ventricular hypertrophy, tissue edema, or reduced diastolic perfusion time) [19]. Different mechanisms of CMD could be involved in different types of CHD.

##### 3.2.2.1 Transposition of the great arteries

The arterial switch operation (ASO) for d-transposition of the great arteries (d-TGA) incorporates translocation of the coronary arteries to allow for adequate perfusion during diastole. This surgical approach leads to early denervation of sympathetic nerves [62]. Multiple studies have demonstrated attenuated coronary flow reserve after administration of the pharmacologic vasodilator therapies, such as nitroglycerin, acetylcholine and adenosine, in primarily asymptomatic patients [[63], [64], [65]]. Interestingly, a study comparing CFR in patients who have undergone ASO versus Ross procedure, demonstrated that the coronary flow reserve was more negatively impacted after ASO when compared with the Ross procedure. These results emphasize that coronary reimplantation alone cannot sufficiently explain the lower coronary flow reserve in ASO patients [65].

##### 3.2.2.2 Single ventricle

Salih et al. studied 15 postmortem heart specimens from hypoplastic heart syndrome (HLHS) patients and demonstrated that hearts with HLHS had significantly reduced capillarization of both left and right ventricles compared with age-matched control hearts, suggesting some fundamental abnormality in coronary capillary network in HLHS [66]. A prospective study utilizing CMR in 119 HLHS patients with Fontan circulation demonstrated compensatory vasodilation of the coronary vasculature at rest allowing them to have similar myocardial blood flow as the control. However, the myocardial blood flow was significantly lower during states of hyperemia compared with the healthy controls [67].

It is postulated that the chronic hypoxemia leading to higher blood viscosity as well as inadvertent sympathetic denervation during Stage I palliation could be the cause of diminished myocardial blood flow during periods of stress. A study conducted on adult patients with cyanotic CHD demonstrated the presence of baseline structural abnormalities including dilation of extramural coronary arteries with increased tortuosity [68]. Furthermore, histology specimens on autopsy demonstrated loss of medial smooth muscle in the dilated coronary arteries with increased medial collagen, fragmented internal elastic lamina and fibrointimal hyperplasia. In addition, viscous erythrocyte perfusate from chronic hypoxemia was thought to cause endothelial shear stress leading to chronic coronary artery dilation. The chronically dilated extramural coronary artery system does lose its ability to further dilate to meet increased oxygen demands during periods of stress. Interestingly, vasodilator levels, such as nitric oxide and vascular endothelial growth factor, were increased secondary to chronic hypoxemia leading to angiogenesis and remodeling of the coronary microcirculation [68]. This remodeling of coronary microcirculation is also thought to maintain the CFR in these patients with HLHS [69].

##### 3.2.2.3 Anomalous left coronary artery from pulmonary artery

Multiple levels of coronary abnormality are expected in anomalous left coronary artery from pulmonary artery (ALCAPA), consisting of secondary collateral vessel formation (arteriogenesis), newly sprouting small vessels (angiogenesis) induced by ischemia, and surgical manipulation of anomalous left coronary artery [70]. In these circumstances, reversible perfusion defect by adenosine stress could potentially occur in the absence of stenosis in epicardial coronary arteries, which may represent microvascular disease [71]. Histologic specimens were obtained and reported on two adults with late diagnosis of ALCAPA after presenting with ventricular arrhythmia. The specimens demonstrated that all layers of the myocardium were affected with variable patchy fibrosis and uniform ischemic changes. The epicardial, endocardial, and middle coronary arteries all demonstrated thickened arteriolar wall with subsequent luminal narrowing secondary to hyalinization and fibrosis of the perivascular stromal tissue). After an ischemic event, there is a known decrease in coronary microvascular perfusion (“slow flow phenomenon”) secondary to microvascular obstruction [72].

##### 3.2.2.4 Pulmonary atresia with intact ventricular septum

Remarkable abnormalities in the capillary distribution have been noted in association with pathological myocardium especially in a thick right ventricle in pulmonary atresia with intact ventricular septum (PA/IVS) [73]. The frequent presence of ventriculo-arterial coronary communication (VCAC), defined as an anomalous connection between the coronary vasculature and the myocardial trabeculae, is a hallmark feature that affects the management of patients with PA/IVS. The presence of VCAC often denotes serious coronary arterial pathology at the site of the connection along with arterial narrowing at the proximal and distal portions. Histologic specimen of a 20-week human fetus has demonstrated intimal thickening and severe adventitial fibrosis predominantly in the subepicardial coronary arteries as well as complete obliterations of coronary arteries with intimal thickening [74]. Overall, VCAC has been found to have a thick outer fibrous layer and thickening of the media and intima of the inner layers and its development has been attributed to a primary coronary vascular anomaly [75].

### 3.3 Myocardial oxygen supply-demand mismatch

Coronary microvascular abnormality can occur independently of the primary vascular endothelial impairment. Myocardial oxygen supply-demand mismatch due to overwhelming biomechanical loads on the working myocardium can aggravate myocardial ischemia by altering coronary microvasculature. This condition has been noted in both primary LV hypertrophy (LVH) (HCM, discussed earlier) and secondary LVH by pressure overloaded heart (i.e., aortic stenosis [AS] and systemic hypertension) [76]. In AS, reduced diastolic time and increased extravascular resistance are the main factors responsible for the reduced CFR [77]. Due to pressure drop across the stenotic aortic valve, systolic acceleration of coronary blood flow decreases while coronary arteries are externally compressed by thick LV myocardium and pressure overload. A reduced diastolic perfusion time during exercise and high diastolic wall stress add to this abnormal coronary flow distribution during exercise or hyperemia. The delayed subendocardial diastolic perfusion after systolic compression is likely responsible for the susceptibility to subendocardial ischemia [78]. These hemodynamic factors can contribute to the pathogenesis of CMD independent of primary endothelial impairment or anatomical alteration of coronary microvasculature. Unlike in HCM, the coronary microvasculature of severe AS patients showed no sign of intramural medial hypertrophy in the intramyocardial arterioles, suggesting the structural changes in coronary microvasculature did not significantly contribute to CMD in AS [79].

### 3.4 Miscellaneous

#### 3.4.1 Genetic

Patients with Turner syndrome with either congenital or acquired heart disease have high rates of all-cause mortality. The patients with Turner syndrome were found to have abnormal myocardial perfusion occurred during childhood and young adulthood [80]. Furthermore, age and obesity were also found to be independent risk factors for coronary microangiopathy. Patients with obesity tend to have thickening of the arterial intima-media leading to increased vasomotor tension, inflammation and endothelial dysfunction which presumably causes CMD [80].

## 4 Diagnosis and treatment of CMD in children

### 4.1 Diagnosis of CMD

Unlike adults who commonly present with signs or symptoms of myocardial ischemia, identification of CMD in children is overall challenging as the incidence of myocardial ischemia or MI is rare in children and may also occur without existing CAD or its risk factors [81,82]. Thus, clinical suspicion from patient history, including substance abuse, tobacco use, and male sex, is essential to effectively diagnose MI in previously healthy adolescents [83]. Dasai et al. reported nine adolescents who developed MI with no known cardiac risk and noted five patients (56 %) had no identifiable epicardial coronary artery obstruction by invasive coronary angiogram with consequent diagnosis of coronary vasospasm [82]. Another study of nine previously healthy adolescents diagnosed with acute myocardial ischemia or infarction revealed no associated identifiable coronary artery abnormality by selective coronary angiography [81]. Both studies indicated high likelihood of CMD as a cause of myocardial impairment in adolescents. However, the distinction from acute myocarditis was not stringent in these studies. Clinical presentation of acute myocarditis in adolescents sometimes resembles that of MI in reference to clinical symptoms (e.g., chest pain) and ECG findings [84].

Multiple diagnostic modalities, noninvasive and invasive, have been utilized for the diagnosis of CMD in symptomatic adults mostly via physiological and functional properties [17,18,85], which may also be available in children. However, direct visualization and morphological assessment of human coronary microvasculature is not possible with current techniques. The experience of these diagnostic studies has been limited in pediatric cardiology practice. High clinical suspicion based upon patients' medical background is essential in making proper diagnosis of CMD in children.

#### 4.1.1 Noninvasive imaging studies

##### 4.1.1.1 Echocardiography

Transthoracic Doppler echocardiography does not directly quantify the capacity of vasodilatation but provides the measurement of flow ratio between maximal diastolic flow in the epicardial arteries at rest and after the treatment with coronary vasodilators (adenosine or dipyridamole). This coronary flow velocity ratio (CFVR) is interpreted as vasodilator capacity of coronary vasculature, identifying the impaired vasodilatory capacity of the microcirculation, which is widely used in clinical practice [86]. Cut -off values of CFVR ≤ 2–2.5 are commonly indicative for impaired coronary microvascular function [87]. However, the measurement requires intense training with many technical pitfalls to obtain reliable values. The interpretation of CFVR requires that epicardial stenosis has been ruled out by anatomical or functional testing [88].

##### 4.1.1.2 Positron Emission Tomography (PET)

Positron emission tomography (PET) is considered the "gold standard" for non-invasive assessment of myocardial blood flow (MBF) and allows CFR by quantification of MBF at rest and during pharmacologically induced maximal hyperemia (ratio of stress/rest MBF) [89,90]. It measures regional MBF of the left ventricle in absolute terms (ml/g/min) but cannot distinguish between structural and functional causes of a decreased hyperemic MBF [88]. In a clinical setting, however, this method is most often subject to limited availability and high cost.

##### 4.1.1.3 Cardiac magnetic resonance (CMR)

Cardiac magnetic resonance with pharmacological stress and gadolinium is used routinely in daily clinical practice for reliable assessment of myocardial ischemia in patients with known or suspected CAD [88]. Thomson et al. recently demonstrated that myocardial perfusion reserve index (MPRI) obtained via CMR, a surrogate of CFR, revealed diagnostic and prognostic value in assessing primary CMD without obstruction of epicardial coronary arteries in middle-aged women with symptomatic angina [91]. Yin et al. performed semi-quantitative evaluation of CMD in hypertrophic cardiomyopathy via CMR first-pass perfusion and late gadolinium enhancement (LGE) imaging and found that the presence of CMD was associated with severity of LGE and extent of hypertrophy [92]. Adenosine stress perfusion CMR imaging was studied in 58 children including 15 with d-TGA after ASO, eight with Kawasaki disease, and five after ALCAPA repair, which demonstrated 100 % negative predictive value in detecting myocardial perfusion defect due to multiple levels of coronary vascular involvement [93]. However, unlike PET that measures absolute myocardial perfusion, semi-quantitative assessment of myocardial perfusion by CMR has some limitation when assessing MPRI, as reduction of MPRI may be due to either increased resting myocardial perfusion or reduction in hyperemic perfusion [88].

#### 4.1.2 Invasive studies

##### 4.1.2.1 Cardiac catheterization and coronary angiography

In adult practice, invasive coronary angiography is widely used for diagnosis of CAD in patients with anginal chest pain and/or dyspnea. Up to 50 % of all patients undergoing elective coronary angiography for the investigation of known or suspected angina, however, have no obstructive epicardial coronary disease [94]. Coronary angiography may be considered incomplete without specifically assessing coronary vasomotion or microcirculation. Historically, slow angiographic contrast movement or coronary slow-flow phenomenon was reported in the patients with anginal chest pain with no obstructive CAD suggestive of CMD [95]. Identification of coronary spasm and/or impaired vasodilation in the absence of epicardial coronary artery obstructions is suggestive of pathological microvascular involvement indicative of CMD in symptomatic patients and helps provide optimum care [96]. Invasive coronary reactivity testing with provocative acetylcholine or ergonovine infusion during cardiac catheterization can be safely performed for the diagnosis of coronary artery spasm [97]. Measurement of CFR and microvascular resistance and vasoreactivity testing to assess coronary spasm are important to guide optimum medication therapy.

## 5 Clinical significance of CMD in children and adolescents

**Fig. 1:**
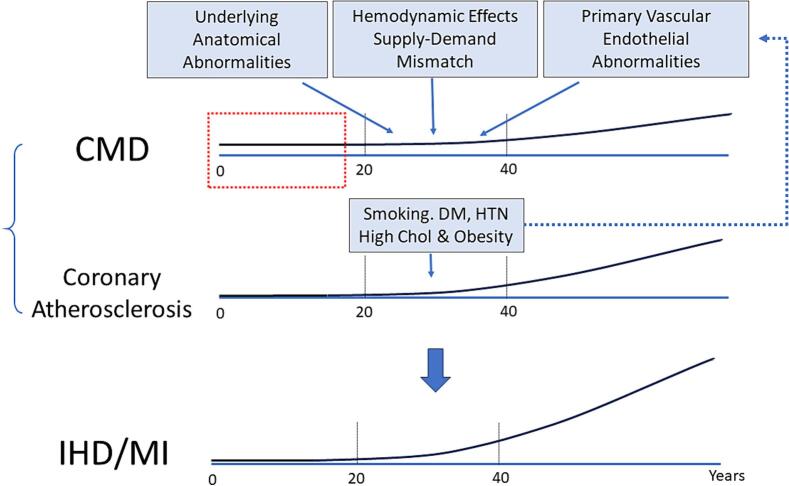
A relationship between cornoary microvascular dysfunction (CMD), coronary atherosclerosis, and ischemic heart disease (IHD)/myocardial infarction (MI). Both CMD and IHD, separately or in combination, contribute IHD and MI. Although CMD occurs during childhood (indicated as red dotted area), it may not be recognized as patients are likely asymptomatic. The incidence of CMD is frequently discussed in association with anginal pain or acute coronary syndrome (postive symptoms of IHD and/or MI). Chol: cholesterol, DM: diabetes mellitus, HTN: hypertension, (For interpretation of the references to color in this figure legend, the reader is referred to the web version of this article.)

The incidence of typical anginal chest pain rarely occurs in children and adolescents as prevalence of coronary atherosclerosis is extremely low in young people. Apart from variable contributing factors of CMD, aging is a fundamental component of the pathogenesis of both CMD and coronary atherosclerosis: Aging is associated with progressive pan-myocardial impairment of coronary vasodilatory capacity due to an increase in minimal microvascular resistance [98]. Age-related impairment of microvascular function impacts the pathophysiology of ischemic heart disease in adult patients. Vascular endothelial dysfunction occurs during the human aging process with deterioration in the balance between vasodilator and vasoconstriction substance produced by endothelium. This process is mainly accompanied by vascular inflammation promoted by age-related oxidative stress independent of traditional risk factors including hypertension, DM, hypercholesterolemia, and smoking [99]. Intrinsic endothelial senescence indicated by shortening of telomeres is associated with aging and hypertension [100]. The incidence of CMD in children and adolescents is low mainly because of its age-dependent nature. In addition, CMD is commonly identified by the presence of angina or acute coronary syndrome. Development of coronary atherosclerosis, another age-dependent factor, is responsible for the patient's symptoms (Fig. 1). This is why CMD is rarely recognized during childhood unless specific studies to assess CFR are performed.

In children and adolescents, CMD can occur due to primary impairment of vascular endothelial cells, abnormal hemodynamic effects inducing myocardial oxygen demand-supply mismatch, and anatomical abnormality of the coronary vasculature. Like in adults, CMD may have a relevant role in the pathogenesis of progressive myocardial dysfunction in children and adolescents by causing occult myocardial ischemia independent of coronary atherosclerosis. The diagnosis of CMD may be essential at an early subclinical stage to prevent consequent adverse cardiac events, including HF, MI, and SCD. However, the diagnosis of CMD in children remains scarce, not because it does not exist, but mainly because the diagnostic coronary vasodilation test is not routinely performed in most pediatric heart centers for asymptomatic children.

How can we manage the CMD after the diagnosis? Are there any effective ways to attenuate or reverse this process? At present, there are no established pharmacological treatment strategies to effectively treat CMD. Hotta et al. studied the rat model, young and old sedentary rats, and examined the effects of exercise training on ventricular diastolic function and coronary microvascular reserve and demonstrated that the exercise improved endothelial function of coronary arterioles, increased coronary blood flow at rest and during exercise and reversed diastolic dysfunction in the aged heart [101]. A six-month exercise training program in older adults, around 60 years of age, with coronary artery disease (cardiac rehabilitation program) was demonstrated to generate positive impact on systemic microvascular endothelial function and oxidative stress [102], suggesting exercise as an effective therapeutic modality for treating CMD in both young and old individuals. In general, exercise exerts many beneficial effects across multiple organ systems to prevent and mitigate cardiometabolic disease, promote health, and increase resilience; exercise enhances coronary angiogenesis to increase myocardial capillary density and improve myocardial energy metabolism [103,104]. However, Rahman et al. studied 85 patients (57 ± 10 years old, 78 % female) who presented with exertional chest pain without obstructive CAD and found that inducible ischemia and inefficient coronary perfusion during exercise in CMD patients, suggesting possible adverse effects by exercise on those with reduced CFR [105]. It is conceivable that different types of CMD, either structural, functional, or combined microvascular abnormality, may result in different outcomes in response to exercise.

## 6 Conclusions

Presence of CMD, defined by diminished CFR in response to pharmacological vasodilation, may indicate increased risk of adverse cardiac events by itself or in combination with existing coronary atherosclerosis, mostly in adults. Because of the paucity of concomitant coronary atherosclerosis and its common risk factors, prognostic significance of CMD in children and adolescents is not necessarily the same as that in adults. However, without active intervention, CMD in childhood will become a serious threat on overall cardiovascular health during adult life. Currently, basic and/or clinical studies regarding CMD in younger people is scarce. Further research endeavors to understand underlying pathobiology and effective treatment are warranted.

## Ethical statement

This article dose not contain any studies with human or animal subjects performed by any of the authors.

## CRediT authorship contribution statement

**Takeshi Tsuda:** Conceptualization, Supervision, Writing – original draft, Writing – review & editing. **Gina Patel:** Writing – original draft.

## Grant Support

N/A.

## Declaration of competing interest

The authors declare that they have no known competing financial interests or personal relationships that could have appeared to influence the work reported in this paper.
